# Low Virus-Specific IgG Antibodies in Adverse Clinical Course and Outcome of Tick-Borne Encephalitis

**DOI:** 10.3390/microorganisms9020332

**Published:** 2021-02-07

**Authors:** Petra Bogovič, Stanka Lotrič-Furlan, Tatjana Avšič-Županc, Miša Korva, Lara Lusa, Klemen Strle, Franc Strle

**Affiliations:** 1 Department of Infectious Diseases, University Medical Centre Ljubljana, Japljeva 2, 1525 Ljubljana, Slovenia; stanka.lotric-furlan@mf.uni-lj.si (S.L.-F.); franc.strle@kclj.si (F.S.); 2 Faculty of Medicine, University of Ljubljana, Vrazov trg 2, 1000 Ljubljana, Slovenia; 3 Institute for Microbiology and Immunology, Faculty of Medicine, University of Ljubljana, Zaloška 4, 1000 Ljubljana, Slovenia; Tatjana.Avsic@mf.uni-lj.si (T.A.-Ž.); misa.korva@mf.uni-lj.si (M.K.); 4 Institute for Biostatistics and Medical Informatics, Faculty of Medicine, University of Ljubljana, Vrazov trg 2, 1000 Ljubljana, Slovenia; lara.lusa@mf.uni-lj.si; 5 Department of Mathematics, Faculty of Mathematics, Natural Sciences and Information Technologies, University of Primorska, Glagoljaška 8, 6000 Koper, Slovenia; 6 Laboratory of Microbial Pathogenesis and Immunology, Division of Infectious Diseases, Wadsworth Center, New York State Department of Health, Albany, 120 New Scotland Ave, Albany, New York, NY 12208, USA; klemen.strle@health.ny.gov

**Keywords:** tick-borne encephalitis, humoral immune response, serum IgG antibodies, course, outcome

## Abstract

Tick-borne encephalitis (TBE) is associated with a range of disease severity. The reasons for this heterogeneity are not clear. Levels of serum IgG antibodies to TBE virus (TBEV) were determined in 691 adult patients during the meningoencephalitic phase of TBE and correlated with detailed clinical and laboratory parameters during acute illness and with the presence of post-encephalitic syndrome (PES) 2–7 years after TBE. Specific IgG antibody levels ranged from below cut-off value (in 32/691 patients, 4.6%), to 896 U/mL (median = 37.3 U/mL). Patients with meningoencephalomyelitis were more often seronegative (24.3%; 9/37) than those with meningoencephalitis (4.7%; 20/428) or meningitis (1.3%; 3/226). Moreover, patients with antibody levels below cut-off had longer hospitalization (13 versus 8 days); more often required intensive care unit treatment (22% versus 8%) and artificial ventilation (71% versus 21%); and had a higher fatality rate (3/32; 9.4% versus 1/659; 0.2%) than seropositive patients. These results were confirmed when antibody levels, rather than cut-off values, were correlated with clinical parameters including the likelihood to develop PES. Low serum IgG antibody responses against TBEV at the onset of neurologic involvement are associated with a more difficult clinical course and unfavorable long-term outcome of TBE, providing a diagnostic and clinical challenge for physicians.

## 1. Introduction

Tick-borne encephalitis (TBE) is a central nervous system disease caused by tick-borne encephalitis virus (TBEV). The majority of human cases are due to 3 subtypes of TBEV, European, Siberian, and Far Eastern, each with a somewhat distinct clinical presentation. The disease caused by the European subtype usually has a biphasic course. The initial phase, which accompanies viremia, manifests with nonspecific febrile illness associated with headaches, myalgias, and fatigue. As viremia decreases these symptoms typically resolve and patients improve. However, after approximately 1-week improvement, patients enter the second phase of TBE which is characterized by neurological involvement; most (50–60%) present with meningitis, 30–40% with meningoencephalitis, and 5–10% with meningoencephalomyelitis. In adults, the fatality rate is up to 2%, approximately 5% of patients have permanent pareses, and at least 1/3 of patients develop post-encephalitic syndrome (PES) (Figure S1). In children, the illness is typically less severe; the most common presentation is meningitis (meningoencephalitis and meningoencephalomyelitis are rare) and children have a better outcome with lower rates of fatality and PES than adults [1,2].

Knowledge of TBE pathogenesis is limited. Clinical manifestations are believed to be the result of a combination of direct viral cytolytic effects and immune-mediated tissue damage, involving both humoral and cellular immune responses. The humoral immune response to TBEV is also important diagnostically. IgM antibodies to TBEV typically appear in serum at the end of the initial phase of TBE and are followed by the appearance of specific IgG antibodies. With the onset of neurologic signs and symptoms (the 2nd phase of TBE), the large majority of patients have both IgM and IgG antibodies to TBEV in serum [3,4,5]. Although TBEV is present in the blood during the first phase of the disease, with the appearance of specific antibodies the virus becomes difficult to detect. During the meningoencephalitic phase, when patients usually seek medical attention and are hospitalized, TBEV is no longer detectable in blood and is only exceptionally found in cerebrospinal fluid (CSF) [1,2]. Thus, in clinical practice, the diagnosis of infection with TBEV is primarily based on clinical signs and symptoms and the demonstration of specific IgM and IgG antibodies in serum. 

Findings from animal models indicate that low TBEV neutralizing antibody levels are associated with higher fatality, implicating humoral immunity in the pathogenesis of TBE [6,7]. However, the corresponding findings in patients with TBE are limited. Gunther et al. reported that among 69 TBE patients, those with encephalitis, a more severe disease course, had significantly lower serum and CSF IgM (but not IgG) levels than those with meningeal symptoms [3]. In contrast, another study of 100 patients with TBE reported a lower prevalence of TBEV IgG antibodies in serum and CSF in patients with severe disease, however, these differences were not significant [8]. Similarly, our previous findings suggest that low TBEV IgG antibody levels during the second phase of TBE are associated with more severe disease, namely that patients with meningitis (relatively mild disease) have higher levels of viral IgG antibodies in serum than those with meningoencephalitis or meningoencephalymyelitis [9,10]. Association between the levels of TBEV antibodies early during the second phase of TBE and the (long-term) outcome of TBE has not been reported.

In the present study, we examined in greater detail the relationship between anti-TBEV IgG antibodies during the early meningoencephalitic phase of TBE and the clinical course and outcome in a large cohort of 691 Central European TBE patients. 

## 2. Patients and Methods 

The study conforms with the World Medical Association Declaration of Helsinki and was approved by the Medical Ethics Committee of the Republic of Slovenia (No 178/2/13, No 152/06/13, No 37/12/13). Each participant provided written informed consent. 

### 2.1. Patients 

During 2007–2012, 717 TBE patients aged ≥18 years who were hospitalized at the Department of Infectious Diseases, University Medical Centre Ljubljana, Slovenia, were enrolled in the study on the course and outcome of TBE. In 2014 (2–7 years after TBE), 420 of these patients were assessed for long-term outcome of TBE. Detailed clinical presentation of TBE and its long-term outcome were reported previously [9,11]. All patients lived in Slovenia and were most likely infected with the European subtype of TBEV; West Nile virus is very rare and no other flaviviruses are known to cause human disease in Slovenia. None of the patients had a history of Japanese encephalitis, dengue, or yellow fever, or vaccination for these diseases. Of the 717 patients, 26 had been vaccinated against TBE and were excluded from the current study. 

Thus, in this study, serum levels of IgG antibodies to TBEV and detailed clinical and laboratory characteristics of TBE were evaluated in 691 patients and correlated with long-term follow-up (2–7 years after TBE) in a subset of 401 patients to determine the role of TBEV-specific IgG antibodies in disease course and outcome. 

### 2.2. Definitions

#### 2.2.1. Definition of Tick-Borne Encephalitis 

TBE was defined as a febrile illness with clinical symptoms and/or signs of meningitis or meningoencephalitis or meningoencephalomyelitis, CSF pleocytosis (>5 × 10^6^ leukocytes/L), and demonstration of recent TBEV infection (presence of serum IgM and IgG antibodies to TBEV). 

Patients with TBE were categorized as having: (i) meningitis when they had only symptoms/signs of meningeal inflammation (fever, headache, the rigidity of the neck, nausea, vomiting); (ii) meningoencephalitis when they had symptoms/signs indicating brain tissue damage (impaired consciousness, concentration, and cognitive function disturbances, tongue fasciculations, tremor of extremities, focal or generalized seizures, etc.) in addition to the findings of meningitis; or (iii) meningoencephalomyelitis when they also had clinical signs of alpha motor neuron injury (flaccid pareses). 

#### 2.2.2. Categorization of the Severity of Tick-Borne Encephalitis 

Severity of the disease was categorized according to clinical diagnosis (meningitis, meningoencephalitis, meningoencephalomyelitis). In all patients except those diagnosed with TBE in 2007 and 2008, the severity of TBE was also evaluated quantitatively using a standardized questionnaire, as reported previously: the presence, intensity, and duration of an individual symptom or sign of TBE were scored on a scale of 1–9 and the absence of a particular symptom or sign as zero; the severity score was defined as the sum of points. In the previous evaluation of this approach, the score ranges 0–8, 9–22, and >22 corresponded to clinically mild (meningitis), moderate, and severe disease, respectively [12].

#### 2.2.3. Tick-Borne Encephalitis-Associated Symptoms 

During hospitalization and at each follow-up visit, patients were asked about the presence of subjective symptoms. Symptoms that newly developed or worsened since the onset of TBE, and which had no other known medical explanation, were interpreted as TBE-associated symptoms [11]. 

#### 2.2.4. Sequelae of Tick-Borne Encephalitis, Post-Encephalitic Syndrome 

For this study, sequelae of TBE were defined as subjective symptoms (headache, fatigue, myalgias, arthralgias, concentration and memory disorders, sleep disorders, emotional lability, dizziness, etc.) fulfilling criteria for TBE-associated symptoms and as objective neurological signs (tremor, ataxia, cranial and spinal nerve pareses, etc.) present at examination 2–7 years after TBE [11]. 

An unfavorable long-term clinical outcome was defined as the existence of PES defined with the presence of ≥2 subjective symptoms fulfilling criteria for TBE-associated symptoms and/or ≥1 objective neurological sign at the last follow-up visit 2–7 years after the acute illness [11].

The severity of PES was interpreted as mild (the presence of 2 symptoms), moderate (the presence of ≥3 symptoms), or severe (the presence of ≥1 objective manifestation with or without subjective symptoms). 

### 2.3. Antibody Determinations 

Antibodies to TBEV were assessed using the Enzygnost^®^ Anti-TBE Virus (IgM, IgG) test (SiemensGmbH, Marburg, Germany) according to the manufacturer’s protocol. During the study period, there were minor changes in cut-off values (6.6–7.0 U/mL). 

### 2.4. Statistical Analyses 

Categorical variables were summarized as frequencies and percentages, and numerical variables as medians and interquartile ranges (IQRs); percentages were reported with 95% confidence intervals (CIs). Univariate and multivariable regression analyses were used to assess the association between levels of anti-TBEV IgG antibodies in serum during the second (meningoencephalitic) phase of TBE and well defined clinical and laboratory characteristics and the outcome of TBE. Additional information on statistical approaches is provided in Supplementary Materials. 

R statistical language was used for all the analyses [13].

## 3. Results

### 3.1. Presentation of Tick-Borne Encephalitis

Basic demographic, clinical, and laboratory characteristics on 691 patients are presented in Table 1. Median duration of the meningoencephalitic phase of illness prior to hospitalization was 4 days, with 62% of patients presenting with meningoencephalitis, 33% with meningitis, and 5% with meningoencephalomyelitis. The median serum level of IgG antibodies to TBEV was 37.3 U/mL but ranged greatly from not detectable to very high (up to 896 U/mL).

#### 3.1.1. Assessment of IgG Tick-Borne Encephalitis Virus Seronegativity with Clinical Course 

To assess the potential association between TBEV antibody responses and severity of TBE, 691 patients were stratified into two groups according to serum TBEV IgG antibody levels above (N = 659, 95.4%) or below (N = 32, 4.6%) the cut-off value (Table 1). All but two patients with IgG antibody levels below the cut-off value had serum IgM antibodies to TBEV at this time point, and in all 32 patients seroconversion was later established. 

The dominant finding was that patients with TBEV antibodies below cut-off had more severe illness. This observation was supported by several parameters. First, patients with meningoencephalomyelitis, the most difficult disease course, had the greatest proportion of IgG seronegative patients (9/37, 24.3%), compared to meningoencephalitis (20/428, 4.7%), or meningitis, a relatively mild illness (3/226, 1.3%). Similarly, patients with IgG TBEV antibodies below cut-off value had higher disease severity scores compared to seropositive patients (18 versus 11 points). Finally, seronegativity was associated with several practical medical care measures indicative of severe course. Compared to seropositive patients, seronegative patients were hospitalized longer (13 versus 8 days) and were more often admitted for >4 weeks (16% versus 1%); they more often needed treatment in the intensive care unit (ICU) (22% versus 8%), remained in ICU longer (17 versus 7 days), and more frequently needed artificial ventilation (71% versus 21%), and for a longer duration (36 versus 5 days). Moreover, of the 4 patients who died during hospitalization 3 were in the seronegative group (3/32; 9.4%) and one was in the seropositive group (1/659; 0.2%). 

Logistic regression analyses substantiated these findings (Table 2). Compared to seropositive patients, seronegative patients were more likely to develop meningoencephalomyelitis (OR = 21.29), they had longer hospitalization (OR = 10.42), were more often admitted to ICU (OR = 3.88), and more frequently required artificial ventilation (OR = 12.37). However, findings in multivariate analysis were not completely congruent. 

#### 3.1.2. Correlation with the Levels of Tick-Borne Encephalitis Virus IgG Antibodies

**Figure 1:**
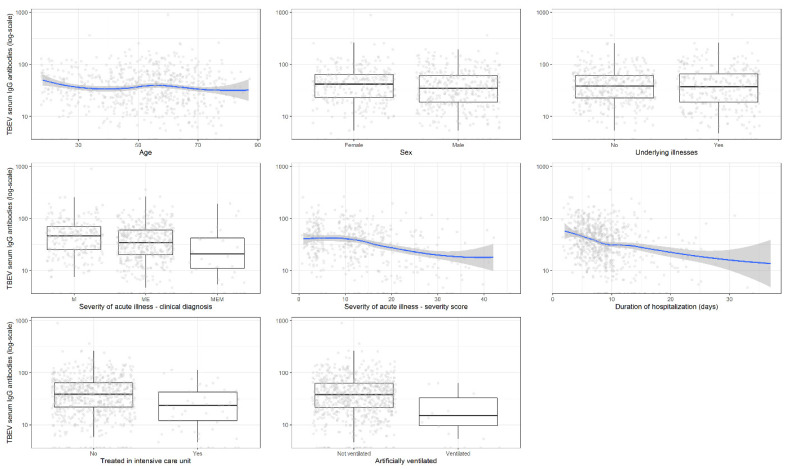
Association of tick-borne encephalitis virus IgG antibody levels in serum (log-scale) with basic demographic and clinical findings including disease severity. Legend: TBEV, tick-borne encephalitis virus.

Because cut-off values are determined for diagnostic purposes, but may not necessarily carry biological utility, we expanded the analyses to test the association between TBEV antibody levels with clinical parameters (Table 2). As with categorical antibody determinations, univariate logistic regression demonstrated that TBEV IgG antibody levels in serum were negatively associated with severity of illness, duration of hospitalization, treatment in ICU, and need for artificial ventilation (Figure 1). In general, multivariable analyses revealed similar results (Table 2). 

**Figure 2:**
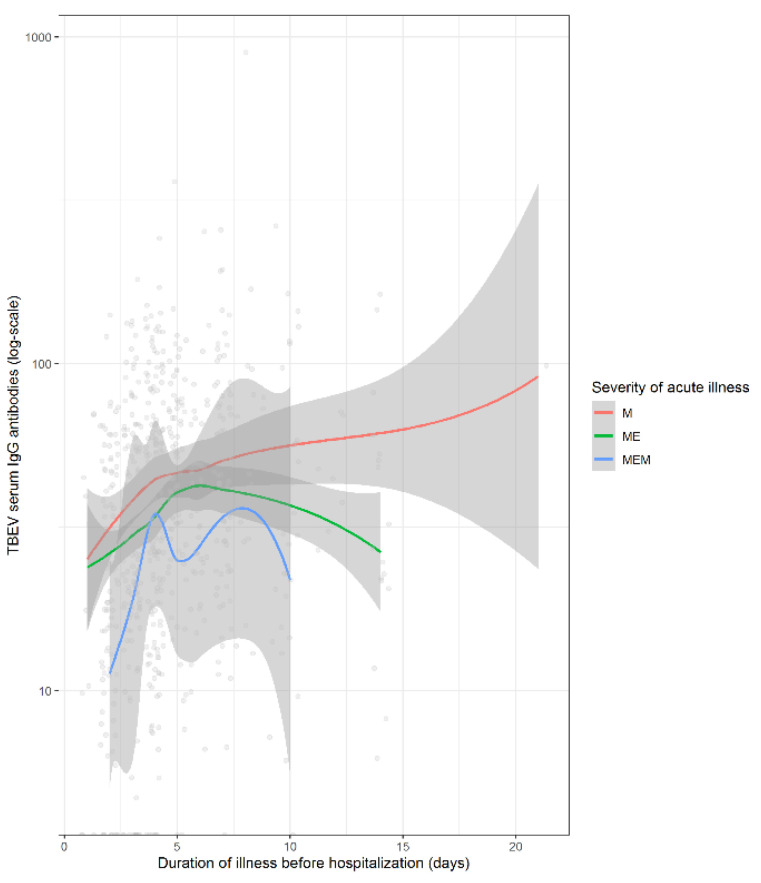
Serum tick-borne encephalitis virus IgG antibody levels (log-scale) according to main clinical manifestation. Legend: TBEV, tick-borne encephalitis virus; M, meningitis; ME, meningoencephalitis; MEM, meningoencephalomyelitis.

Comparison of antibody levels revealed that patients with meningoencephalomyelitis and meningoencephalitis have lower levels of the antibodies than those with meningitis throughout the course of the meningoencephalitic phase of illness (Figure 2). Collectively, these data imply that some patients do not develop robust TBEV antibody responses and that insufficient responses (not only those beyond the cut-off) are associated with a more difficult disease course. 

### 3.2. Long-Term Outcome of Tick-Borne Encephalitis 

Because of the association between low antibody levels and more severe acute illness, we then examined the impact of early antibody responses on the long-term outcome of TBE. This information was available for 401 patients who were evaluated 2–7 years after TBE; 267 (66.6%) had favorable outcomes whereas 134 (33.4%) had PES. The demographic, clinical, and laboratory characteristics at the time of TBE are shown in Table 3. 

#### 3.2.1. Tick-Borne Encephalitis Virus IgG Antibodies below or above Cut-Off Value

In 13 (3.2%) patients, serum TBEV IgG antibodies were below the cut-off value early in the second phase of TBE; 12 had IgM antibodies to TBEV in serum at this time point, and all of them later seroconverted. The proportion of IgG TBEV seronegative patients tended to be higher in those who later developed PES (6/134, 4.5%) than in those who had a favorable outcome (7/267, 2.6%; *p* = 0.373). Furthermore, when stratified according to PES severity, the proportion of patients with IgG antibodies below cut-off was highest in those with severe PES (2/17, 11.8%, 95% CI 1.5–36.4), intermediate in those with moderate PES (1/19, 5.3%, 95% CI 0.1–33.1), and the lowest in those with mild PES (3/98, 3.1%, 95% CI 0.6–8.7). Consistent with these findings, univariate logistic regression showed a positive association (OR = 1.75, 95% CI 0.58–5.27; *p* = 0.308) toward a higher prevalence of PES in seronegative patients (Table 4). 

#### 3.2.2. Levels of Tick-Borne Encephalitis Virus IgG Antibodies in Patients with Post-Encephalitic Syndrome

Univariate logistic regression showed that lower levels of serum IgG antibodies to TBEV were associated with a higher likelihood of PES 2–7 years after TBE (OR = 0.78, 95% CI: 0.61–0.98; *p* = 0.032). The same trend was observed when the analysis was adjusted for other possible confounding variables (OR = 0.83, 95% CI: 0.64–1.06; *p* = 0.136). Detailed information is provided in Table 4.

## 4. Discussion

In the current study, we investigated the associations between TBEV IgG antibody responses during the early meningoencephalitic phase of TBE and the clinical course and long-term outcome of TBE. Patients who were previously vaccinated against TBE were excluded because they develop very high levels of serum IgG antibodies to TBEV during the first days of the neurologic involvement [1,2,14], which would interfere with the interpretation of the findings. 

Previous studies reported that higher CSF leukocyte counts [8,9,15], higher serum CRP concentrations [9,16], and male sex [17] were associated with more severe TBE; but the association of the three parameters with lower TBEV antibody levels, as uncovered in the present study, has not been described. Furthermore, previous studies suggested association of lower levels of (neutralizing) antibodies to TBEV with a more severe course of TBE [3,8,9,10] while in the present study regression analyses revealed that lower levels of IgG serum antibodies to TBEV are associated with more severe illness (according to clinical diagnosis and severity score), longer duration of hospitalization, treatment in ICU, and need for artificial ventilation. In contrast, higher serum antibody levels are associated with a longer duration of the illness prior to testing (Table 2). Yet, the associations with more severe illness remain at each time point throughout the course of the meningoencephalitic phase of illness (days 0–20) demonstrating that the duration of illness prior to hospitalization was not the reason for the low antibody responses and severe disease. Furthermore, we established that lower antibody responses are associated not only with the more severe clinical course but also with the unfavorable outcome of TBE which was statistically significant in univariate but not in multivariate analyses. 

In the present study, 95% of patients hospitalized for TBE had serum TBEV IgG antibodies during the first days of neurologic involvement, however, 5% of patients were seronegative (below assay cut-off). Although the reasons for the lack of antibody response is not clear as the majority of these patients (90%) did not have a known immunocompromising condition, comparison with seropositive patients resulted in several novel findings of practical value. First, IgG seronegativity was associated with more severe disease: the ORs for IgG seronegativity were 3.2-fold higher in patients with meningoencephalitis and 21.3-times higher in those with meningoencephalomyelitis compared to patients with meningitis. The strength of this finding was supported by analysis according to severity score, as well by multiple direct parameters commonly used for clinical assessment, including longer hospitalization (1.6×), hospitalization for >4 weeks (13×), need for ICU treatment (2.8×), longer ICU stay (2.6×), need for artificial ventilation (3.4×) and a longer duration (7.9×) of the ventilation, and higher fatality rate (47×). Moreover, TBEV IgG seronegativity during TBE was associated with the presence of PES years after the infection. Although not all of these differences reached statistical significance, similar findings were observed when clinical parameters were assessed according to TBEV IgG antibody levels rather than categorical cut-offs, and in each instance, the trends were the same and carried the same message: lower antibody levels are associated with more severe illness including the greater probability of death (Table 2). 

The findings that lower levels of IgG antibodies to TBEV (determined early in the course of meningoencephalitic phase of TBE) are associated with more severe acute illness, and with more chances to develop PES, suggest the involvement of humoral immune response in the pathogenesis of TBE, presumably by controlling TBEV infection. We do not know if specific IgG antibodies were directly associated with the variables or were a reflection of altered reactivity of some other factors. The development of antibodies to an infecting agent, including TBEV, depends primarily on the proper function of B cells. However, the cellular aspects of B cell response, including phenotype and activation status, as well as the overall B cell role in TBE pathogenesis have not been delineated [18].

The study has several limitations. The evaluation of serum antibodies to TBEV was performed using a commercial ELISA diagnostic assay (Enzygnost^®^ Anti-TBE Virus (IgM, IgG) test; SiemensGmbH). While concordant results were achieved using the different tests in a separate study [19], a recent report which directly compared four commercial IgG ELISAs revealed pronounced discrepancies, predominantly in the detection of antibodies after vaccination [20]. Although in the present study patients with TBE who were previously vaccinated against this disease were excluded, the limitation is that we did not consider other assays. Furthermore, we also did not assess the presence of neutralizing antibodies but the concordance of diagnostic antibodies (as determined in ELISA) and protective (neutralizing) antibodies is very high (98%) [21,22,23]. Moreover, antibody function (neutralization) is likely to be particularly important in patients who develop severe disease or in vaccinated individuals who develop TBE despite high TBEV IgG antibody levels. In contrast, the focus of our study was to assess low antibody levels in disease presentation, a situation in which the major variable is the lack of antibody response rather than antibody function. Still, one cannot rule out the possibility that functional differences may contribute to disease severity. 

A partial explanation for the finding that IgG seronegativity was associated with more severe disease could be that the diagnosis of TBE in some patients is discounted a priori because of the absence of specific IgG antibodies in serum and that the ratio of neglected TBE cases is higher in patients with mild illness than in those with the severe disease for whom a more comprehensive diagnostic workup in usually performed [12,24]. The likelihood of such diagnostic bias in the present study is probably negligible because all patients were diagnosed at a single institution with extensive TBE experience, and because of a strict diagnostic approach that included TBE serological testing in all patients with aseptic meningitis and retesting in a few days in case of diagnostic uncertainty. 

Another limitation of this study is that the results are based on TBE patients infected with the European subtype of TBEV and may not apply to the disease caused by other virus subtypes. In addition, our findings in adults need to be verified in children in whom antibody dynamics may be somewhat different. Moreover, as mentioned above, much remains to be learned about the mechanisms underlying B cell function as well as antibody function in TBE. Although the study indicates antibody levels as being important, the information on antibody function (e.g., neutralization) and cellular immunity remain to be elucidated. This is particularly true since there is a temporal dissociation between peak viremia, peak antibody production, and the clinical presentation of the disease. However, the goal of this study was to provide practical information for clinicians to aid the management and care of patients with TBE.

In conclusion, our study revealed that deficient systemic IgG antibody responses to TBEV are associated with more difficult clinical course and with an unfavorable long-term outcome of TBE as evidenced by the greater prevalence of meningoencephalomyelitis, longer hospitalization and ICU treatment, and higher prevalence of PES years after TBE. These data underscore the importance of robust humoral immune response in the pathogenesis of TBE. Moreover, the findings offer practical information for physicians who need to be aware that TBEV IgG levels below the cut-off value may pose challenges for diagnosis as well as for managing the more severe acute illness and less favorable outcome of patients with TBE.

## Figures and Tables

**Table 1:** Basic demographic, clinical, and laboratory data on 691 adults with tick-borne encephalitis.

Characteristic	All Patients (No = 691)	Patients with Positive IgG Levels to TBEV (No = 659)	Patients with Negative IgG Levels to TBEV (No = 32)
	Number (%, 95% CI) or Median (IQR)		
Male sex	391 (56.6, 52.8–60.3)	369 (56.0, 52.1–59.8)	22 (68.8, 50.0–83.4)
Age (years)	54 (41–64)	53 (41–63)	55.5 (40.75–65)
Underlying illnesses	300 (43.4, 39.7–47.2)	285 (43.2, 39.4–47.1)	15 (46.9, 29.1–65.3)
Severe underlying immunocompromised condition ^a^	12 (1.7, 0.9–3.0)	9 (1.4, 0.6–2.6)	3 (9.4, 2.0–25.0)
Duration of illness before hospitalization (days) ^b^	4 (3–6) (DA 631)	4 (3–6.75) (DA 602)	3 (2–3) (DA 29)
Biphasic course of illness	418/680 (61.5, 57.7–65.2)	394/648 (60.8, 56.9–64.6)	24 (75.0, 56.6–88.5)
Clinical presentation			
Meningitis	226 (32.7, 29.2–36.3)	223 (33.8, 30.2–37.6)	3 (9.4, 2.0–25.0)
Meningoencephalitis	428 (61.9, 58.2–65.6)	408 (62.0, 58.1–65.6)	20 (62.5, 43.7–78.9)
Meningoencephalomyelitis	37 (5.4, 3.8–7.3)	28 (4.2, 2.8–6.1)	9 (28.1, 13.8–46.8)
Severity of illness (severity score)	12 (5–18) (DA 449)	11 (4–18) (DA 429)	18 (13–22.75) (DA 20)
Treatment in intensive care			
unit; number	53 (7.7, 5.8–9.9)	46 (7.0, 5.2–9.2)	7 (21.9, 9.3–40.0)
duration (days)	8 (4–10)	7 (4–9)	17 (7.5–73)
Artificial ventilation; number	15 (2.2, 1.2–3.6)	10 (1.5, 0.7–2.8)	5 (15.6, 5.3–32.8)
duration (days)	7 (3.5–22.5)	4.5 (3.25–7)	35 (10–38)
Hospitalization (days)	8 (6–10)	8 (6–10)	13 (9–17.25)
hospitalization > 4 weeks; number	13 (1.9, 1.0–3.2)	8 (1.2, 0.5–2.4)	5 (15.6, 5.3–32.8)
Death in acute phase of illness (during hospitalization)	4 (0.6, 0.2–1.5)	1 (0.2, 0–0.8)	3 (9.4, 2.–25.0)
Blood leukocyte count (×10^9^ cells/L)	10.2 (8.48–12.4) (DA 684)	10.2 (8.5–12.4) (DA 652)	9.45 (7.625–12.025)
Serum CRP level (mg/L)	7 (3–16) (DA 683)	7 (3–16) (DA 651)	4.5 (3–8.75)
CSF laboratory findings			
Leukocyte count (×10^6^ cells/L)	85 (42–153)	84 (42–149)	184 (56.5–249.25)
Protein concentration (g/L)	0.69 (0.53–0.91) (DA 690)	0.7 (0.5325–0.9175) (DA 658)	0.65 (0.5375–0.805)
Glucose concentration (mmol/L)	3.0 (2.7–3.3) (DA 685)	3.0 (2.7–3.3) (DA 653)	2.9 (2.7–3.3)
Level of TBEV IgG antibodies (U/mL)	37.3 (19.7–64.2)	38 (21.25–62.2)	NA
Concomitant Lyme neuroborreliosis ^c^	21/635 (3.3, 2.1–5.0)	21/605 (3.5, 2.2–5.3)	0 (0, 0–10.9)
Positive *B. burgdorferi* sensu lato IgG antibodies in serum ^d^	61/629 (9.7, 7.5–12.3)	58/599 (9.7, 7.4–12.3)	3/30 (10.0, 2.1–26.5)

^a^ Active malignant disease, receiving immunosuppressive treatment or biologic drugs. ^b^ In patients with biphasic course of illness counted from the beginning of the second (meningoencephalitic) phase until hospitalization. ^c^ Demonstration of *Borrelia burgdorferi* sensu lato infection of the central nervous system by isolation of *B. burgdorferi* sensu lato from CSF or intrathecal synthesis of *B. burgdorferi* sensu lato specific IgG or IgM antibodies. IgM antibodies to outer surface protein C (OspC) and variable-like sequence expressed (VlsE) antigen, and IgG antibodies to VlsE borrelial antigens, were determined in serum and CSF using an indirect chemiluminescence immunoassay (LIAISON, Diasorin, Italy) and results interpreted according to the manufacturer’s instructions. ^d^ The only marker of borrelial infection. TBEV, tick-borne encephalitis virus; CI, confidence interval; IQR, interquartile range; DA, data available (number of patients with available data; information given only in case of incomplete data); CRP, C-reactive protein; CSF, cerebrospinal fluid; NA, not applicable.

**Table 2:** Covariates representing acute illness associated with the negative IgG antibodies/levels of IgG antibodies to tick-borne encephalitis virus in the serum of patients early during the meningoencephalitic phase of tick-borne encephalitis.

Covariate	Negative IgG Antibodies to TBEV		Levels of IgG Antibodies to TBEV	
	Univariate Analysis OR (95% CI); *P*	Multivariable Analysis OR (95% CI); *P*	Univariate Analysis EC ^a^ (95% CI); *P*	Multivariable Analysis EC ^a^ (95% CI); *P*
Age	1.03 (0.83–1.29); 0.791 ^b^	0.89 (0.83–0.93); 0.284 ^b^	0.96 (0.92–1.01); 0.151	1.01 (0.95–1.06); 0.850
Male sex	1.68 (0.82–3.71); 0.162	1.50 (1.26–1.72); 0.332	0.82 (0.69–0.95); **0.016**	0.84 (0.72–0.97); **0.024**
Underlying illnesses	1.16 (0.57–2.35); 0.675	0.89 (0.72–1.02); 0.661	0.95 (0.80–1.10); 0.543	1.09 (0.91–1.26); 0.330
Duration of illness before hospitalization ^c^	0.65 (0.50–0.82); **<0.001**	0.59 (0.49–0.64); **0.019**	1.58 (1.30–1.87); **<0.001** ^b^	1.25 (1.03–1.47); **<0.001** ^b^
Serum CRP level ^d^	0.82 (0.52–1.13); 0.256	0.58 (0.52–0.86); **0.001**	1.53 (1.24–1.81); **<0.001** ^b^	1.41 (1.16–1.66); **<0.001** ^b^
CSF leukocyte count ^d^	1.58 (1.29–1.93); **<0.001**	1.65 (1.26–1.74); **<0.001**	1.03 (0.87–1.20); **<0.001** ^b^	0.87 (0.73–1.01); **0.002** ^b^
CSF protein concentration ^d^	0.93 (0.60–1.33); 0.722	0.86 (0.77–0.93); 0.417	1.02 (0.93–1.10); 0.706 ^b^	1.03 (0.95–1.12); 0.466 ^b^
Severity of acute illness				
Clinical assessment ^e^				
Meningoencephalitis	3.20 (1.15–12.16); **0.024**	NA	0.70 (0.59–0.82); **<0.001**	NA
Meningoencephalomyelitis	21.29 (6.33–89.38); **<0.001**	NA	0.27 (0.17–0.36); **<0.001**	NA
Quantitative assessment ^f^	2.54 (1.75–3.71); **<0.001**	1.18 (1.02–1.30); 0.751	0.67 (0.61–0.73); **<0.001** ^b^	0.89 (0.78–1.0); 0.063 ^b^
Duration of hospitalization	10.42 (0–29.64); **<0.001**	8.51 (0.00–21.7); **0.018**	0.63 (0.55–0.70); **<0.001** ^b^	0.71 (0.60–0.82); **<0.001** ^b^
Treated in ICU	3.88 (1.53–8.86); **0.006**	0.62 (0.43–0.77); 0.378	0.46 (0.32–0.59); **<0.001**	0.94 (0.62–1.26); 0.747
Artificially ventilated	12.37 (3.84–36.17); **<0.001**	1.70 (1.09–2.62); 0.690	0.21 (0.10–0.33); **<0.001**	0.63 (0.23–1.04); 0.160

^a^ The estimated ratio evaluates the ratio of level of IgG antibodies to TBEV (for categorical variables: for patients in the category relative to those of the reference category; for numerical variables: for patients with the value of the covariate at third quartile relative to those at first quartile). ^b^ The variable was modeled using restricted cubic splines; the reported estimates compare patients with values of the variable equal to third quantile relative to patients with value at 1st quartile; the *p*-values are overall *p*-values, obtained using type-2 ANOVA. The interquartile ranges were: Age: 41–64 years, Duration of illness before hospitalization: 3–6 days, Serum CRP level: 3–16 mg/L, CSF leukocyte count: 42–153 × 10^6^ cells/L, CSF protein concentration: 0.535–0.91 g/L, Duration of hospitalization: 6–10 days). ^c^ In patients with a biphasic course of illness counted from the beginning of the second (meningoencephalitic) phase until hospitalization. ^d^ Laboratory findings determined at the initial examination of the meningitic/meningoencephalitic phase of TBE were compared. ^e^ Clinical assessment was not included in multivariable models due to its strong association to quantitative assessment^. f^ Data available for 401 patients, missing data were imputed (see methods for details). For numerical covariates, the third and first quartiles are compared. Remark: Using the Firth regression method, 95% CIs not encompassing 1 does not need to be associated with *p* < 0.05. TBEV, tick-borne encephalitis virus; OR, odds ratio; CI, confidence interval; EC, estimated coefficient; CRP, C-reactive protein; CSF, cerebrospinal fluid; NA, not applicable; ICU, intensive care unit.

**Table 3:** Basic demographic, clinical, and laboratory data on 401 adult patients with tick-borne encephalitis according to the outcome of the disease 2–7 years later.

Characteristic	All Patients (No = 401)	Patients with Favorable Outcome (No = 267)	Patients with PES (No = 134)
	Number (%, 95% CI) or Median (IQR)		
Age (years)	55 (43–63)	55 (40.5–64)	54.5 (44–62)
Male sex	215 (53.6, 48.6–58.6)	149 (55.8, 49.6–61.9)	66 (49.3, 40.5–58.0)
Underlying illnesses	179 (44.6, 39.7–49.7)	113 (42.3, 36.3–48.5)	66 (49.3, 40.5–58.0)
CSF leukocyte count (×10^6^ cells/L)	80 (40–139)	80 (40–133)	88.5 (39–153.5)
CSF protein concentration (g/L)	0.69 (0.54–0.92)	0.68 (0.54–0.925)	0.71 (0.55–0.92)
Severity of acute illness			
Clinical presentation			
Meningitis	126 (31.4, 26.9–36.2)	90 (33.7, 28.1–39.7)	36 (26.9, 19.6–35.2)
Meningoencephalitis	257 (64.1, 59.2–68.8)	172 (64.4, 58.4–70.2)	85 (63.4, 54.7–71.6)
Meningoencephalomyelitis	18 (4.5, 2.7–7.0)	5 (1.9, 0.6–6.4)	13 (9.7, 5.3–16.0)
Quantitative assessment (severity score)	12 (5–18)	11 (4–17.75)	13 (5.5–19.5)
Level of TBEV IgG antibodies (U/mL)	38.4 (19.6–62.8)	41.9 (21.9–63.4)	34.6 (17.2–61.15)
Negative IgG antibodies to TBEV	13 (3.2, 1.7–5.5)	7 (2.6, 1.1–5.3)	6 (4.5, 1.7–9.5)
Concomitant Lyme neuroborreliosis ^a^	8/369 (2.2, 0.9–4.2)	4/247 (1.6, 0.4–4.1)	4/122 (3.3, 0.9–8.2)

^a^ Demonstration of *Borrelia burgdorferi* sensu lato infection of the central nervous system by isolation of *B. burgdorferi* sensu lato from CSF or intrathecal synthesis of *B. burgdorferi* sensu lato specific IgG or IgM antibodies. IgM antibodies to outer surface protein C (OspC) and variable-like sequence expressed (VlsE) antigen, and IgG antibodies to VlsE borrelial antigens, were determined in serum and CSF using an indirect chemiluminescence immunoassay (LIAISON, Diasorin, Italy) and results interpreted according to the manufacturer’s instructions. PES, post-encephalitic syndrome; CI, confidence interval; IQR, interquartile range; CSF, cerebrospinal fluid; TBEV, tick-borne encephalitis virus.

**Table 4:** Association of covariates representing acute illness with the post-encephalitic syndrome.

Covariate	Post-Encephalitic Syndrome ^a^	
	Univariate Analysis OR (95% CI); *P* ^b^	Multivariable Analysis OR (95% CI); *P* ^b^
Age ^c^	0.47 (0.18–0.76); **0.003**	0.38 (0.12–0.64); **0.010**
Male sex	0.77 (0.51–1.16); 0.215	0.71 (0.46–1.10); 0.130
Underlying illnesses	1.32 (0.87–2.00); 0.188	1.46 (0.90–2.39); 0.129
CSF leukocyte count ^d^	1.10 (0.90–1.34); 0.340	1.04 (0.83–1.30); 0.739
CSF protein concentration ^d^	1.16 (0.95–1.41); 0.148	1.09 (0.87–1.36); 0.470
Severity of acute illness		
Clinical presentation ^e^		
Meningoencephalitis	1.23 (0.78–1.97); 0.381	NA
Meningoencephalomyelitis	6.09 (2.20–19.13); **<0.001**	NA
Quantitative assessment	1.41 (1.09–1.83); **0.008**	1.31 (0.99–1.75); 0.056
(severity score)		
Serum IgG antibodies to TBEV		
Levels ^d^ Log-IgG	0.78 (0.61–0.98); **0.032**	0.83 (0.64–1.06); 0.136
Negative IgG ^d^	1.75 (0.58–5.27); 0.308	NA
Concomitant LNB ^f^	2.02 (0.51–7.94); 0.303	1.94 (0.47–8.04); 0.350

^a^ Postenecphalitic syndrome defined with the presence of ≥2 subjective symptoms fulfilling criteria for tick-borne encephalitis associated symptoms and/or ≥1 objective neurological sign at the last follow-up visit 2–7 years after the acute illness (please see Supplementary Material). ^b^ Overall *p*-value is given. For numerical covariates, the third and first quartiles are compared. ^c^ Modelled using restricted cubic splines- the reported OR compares the odds for post-encephalitic syndrome for patients with age at 3rd quartile (64 years) versus those with age at first quartile (41 years). ^d^ Laboratory findings determined at the initial examination of the meningitic/meningoencephalitic phase of tick-borne encephalitis. ^e^ Meningitis is the reference category, the overall *p*-value in the univariate analysis is 0.002. ^f^ Demonstration of *Borrelia burgdorferi* sensu lato infection of the central nervous system by isolation of *B. burgdorferi* sensu lato from CSF and/or intrathecal synthesis of *B. burgdorferi* sensu lato specific IgG or IgM antibodies. IgM antibodies to outer surface protein C (OspC) and variable-like sequence expressed (VlsE) antigen, and IgG antibodies to VlsE borrelial antigens, were determined in serum and CSF using an indirect chemiluminescence immunoassay (LIAISON, Diasorin, Italy) and results interpreted according to the manufacturer’s instructions. OR, odds ratio; CI, confidence interval; CSF, cerebrospinal fluid; NA, not applicable; TBEV, tick-borne encephalitis virus; LNB, Lyme neuroborreliosis.

## Data Availability

The data presented in this study are available on request from the corresponding author. The data are not publicly available due to the protection of patient personal information.

## Supplementary Materials

Statistical Analyses, https://www.mdpi.com/2076-2607/9/2/332/s1, Figure S1: The course and outcome of tick-borne encephalitis, Figure S2. Estimated probability of post-encephalitic syndrome according to age.
