# Supplementary material for: Low Virus-Specific IgG Antibodies in Adverse Clinical Course and Outcome of Tick-Borne Encephalitis
Source: Microorganisms. 2021 Feb 7;9(2):332. doi: 10.3390/microorganisms9020332 (PMC7914885; doi:10.3390/microorganisms9020332)
Supplement: Supplementary file 1 [file microorganisms-09-00332-s001.pdf]

Supplementary materials

Statistical analyses

Categorical variables were summarized as frequencies and percentages, and numerical variables as medians and interquartile ranges (IQRs); percentages were reported with 95% confidence intervals (CIs).

Presentation of tick-borne encephalitis

We employed univariate and multivariable regression to assess the association between levels of anti- TBEV IgG antibodies in serum in the early second (meningoencephalitic) phase of TBE and 14 well defined clinical and laboratory parameters. Compared covariates were selected using expert opinion (P.B. and F.S.), independent from the observed outcomes. Because the albumin and IgG quotient had a rather large proportion of missing values (175/691), and were strongly associated to protein concentration in CSF (Spearman's rho >0.85), only CSF protein concentration was used in the analyses. The 12 covariates used in univariate and multivariable models are reported in Table 2. The outcome variables (serum levels of IgG antibodies to TBE virus) were log-transformed. The numerical covariates that in exploratory data analysis showed a univariate non-linear association with the serum levels of IgG antibodies (such as age - Supplementary fig. 2) were modelled using restricted cubic splines (RCS) [1]. Missing values were imputed using mean values for numerical variables and mode for categorical variables; for severity score, the imputation was conditional on the clinical category of severity (meningitis, meningoencephalitis or meningoencephalomyelitis), which was available for all patients. To assess if the use of this simple imputation method had an impact on the results, multivariable imputation by chained equations was also employed [2].

Numerical covariates were standardized according to the IQRs. Results were summarized with estimated coefficients (ECs), 95% CIs, and P values based on the Wald test. P values <0.05 were considered statistically significant. The ECs for the variables modelled with RCS, which are difficult to interpret, were summarized as the estimated average difference in the outcome comparing two subjects whose value of the covariate are equal to the observed 3rd and 1st quartile; the interpretation of the reported coefficients is therefore similar as for the other numerical variables with IQR standardization.

Outcome of tick-borne encephalitis

Since the information on the long-term outcome was missing for one third of patients and the data on outcome refer to a later time point compared to the other covariates used in the previous models, the possible explanatory nature of the initial models expanded with disease outcome data would be lost. This is why we decided not to include the outcome variables in the original multiple regression model, but look at them separately. Yet, for the assessment of the association between levels of anti TBE virus IgG antibodies in serum and outcome of TBE (presence of post-encephalitic syndrome) 2–7 years after acute illness we employed similar statistical approaches, i.e., univariate and multivariable regression; and also here the compared covariates were selected using expert opinion (P.B. and F.S.), independent from the observed outcomes. However, since the number of cases assessed for the outcome of TBE was lower than the number of cases assessed for the characteristics of acute illness, we diminished the number of selected covariates from 12 to 10.

References

1. Harrell FE Jr, Lee KL, Pollock BG. Regression models in clinical studies: determining relationships between predictors and response. J Natl Cancer Inst 1988; 80:1198–202. doi: 10.1093/jnci/80.15.1198.
2. Van Buuren S. Flexible Imputation of Missing Data. 2nd ed. Boca Raton, FL: CRC Press; 2018.

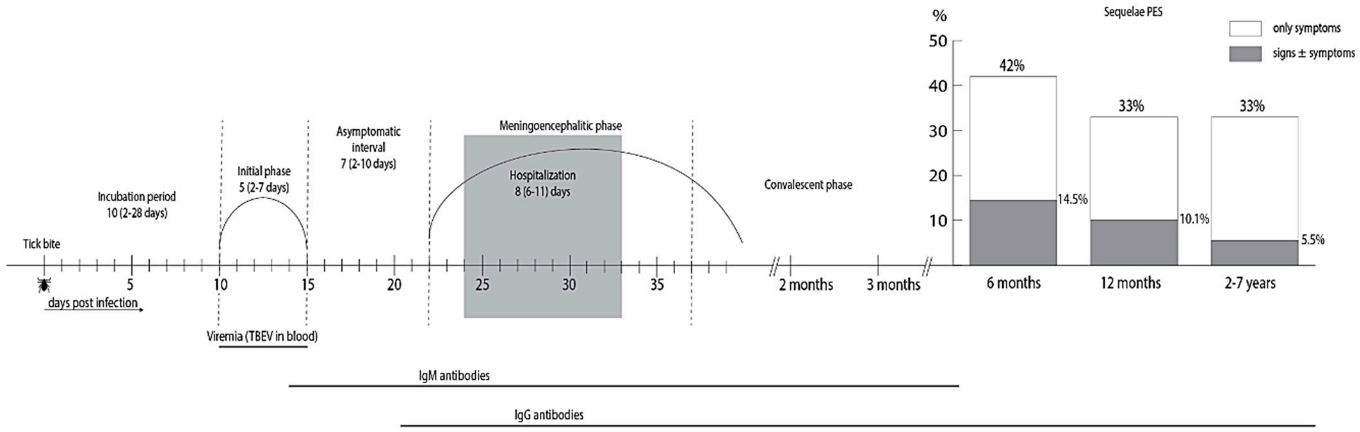

Figure S1. The course and outcome of tick-borne encephalitis. Legend: TBEV, tick-borne encephalitis virus; PES, post-encephalitic syndrome.

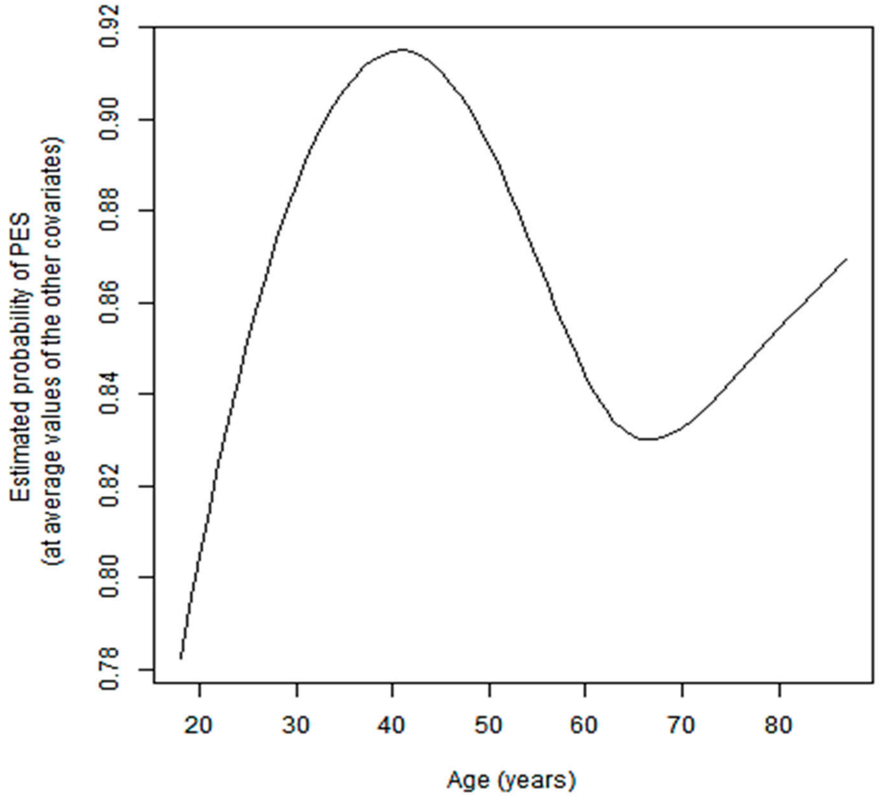

Figure S2. Estimated probability of post-encephalitic syndrome according to age. Legend: PES, post-encephalitic syndrome.
